# Duodenal Organoids From Metabolic Dysfunction-Associated Steatohepatitis Patients Exhibit Absorptive and Barrier Alterations

**DOI:** 10.1016/j.gastha.2024.100599

**Published:** 2024-12-12

**Authors:** Alia Hadefi, Morgane Leprovots, Gilles Dinsart, Maryam Marefati, Marjorie Vermeersch, Daniel Monteyne, David Pérez-Morga, Anne Lefort, Frédérick Libert, Laurine Verset, Claire Liefferinckx, Christophe Moreno, Jacques Devière, Eric Trépo, Marie-Isabelle Garcia

**Affiliations:** 1 IRIBHM, Jacques E. Dumont, Faculty of Medicine, Université Libre de Bruxelles ULB, Brussels, Belgium; 2 Department of Gastroenterology, Hepatopancreatology, and Digestive Oncology, CUB Hôpital Erasme, Hôpital Universitaire de Bruxelles, Université Libre de Bruxelles, Brussels, Belgium; 3 Laboratory of Experimental Gastroenterology, Université Libre de Bruxelles, Brussels, Belgium; 4 Center for Microscopy and Molecular Imaging, Université Libre de Bruxelles (ULB), Charleroi, Belgium; 5 BRIGHTcore ULB-VUB and Institute of Interdisciplinary Research in Human and Molecular Biology (IRIBHM), Université Libre de Bruxelles, Brussels, Belgium; 6 Institut Jules Bordet, Hôpital Universitaire de Bruxelles, Centre d’Anatomie pathologique, rue Meylermeersch, Brussels, Belgium

**Keywords:** MASH, Stem Cells, Cell Adhesion, Organoids, Intestine

## Abstract

**Background and Aims:**

Metabolic dysfunction-associated steatohepatitis (MASH) is a progressive liver disease that can lead to fibrosis, cirrhosis, and hepatocellular carcinoma. Though MASH is closely tied to metabolic risk factors, the underlying pathogenic mechanisms remain scarcely understood. Recent research has emphasized the importance of the gut-liver axis in its pathogenesis, an aspect less explored in human studies. Here, we investigated whether the duodenal epithelium of MASH patients could exhibit intrinsic dysfunctions.

**Methods:**

Duodenal epithelial organoids were generated from 16 MASH patients and 14 healthy controls. Biopsies and patient-derived organoid transcriptomes were then analyzed to evaluate if specific intestinal pathways were differentially modulated in MASH subjects. Functional assays were performed to assess the duodenal epithelial absorptive potential and barrier functionality.

**Results:**

Organoid formation efficiency was similar between control-derived duodenal epithelial organoids and MASH-derived duodenal epithelial organoids (MDEOs) (71% and 69%, respectively). Despite global heterogeneity in growth patterns, MDEOs frequently exhibited cystic spheroid morphology. MDEOs displayed altered digestive potential associated with reduced mature absorptive cell fate, but they retained their lipid metabolic capacity, possibly mediated by lipid oxidation in stem/progenitor cells. Additionally, MDEOs misexpressed components of tight and adherens junctions and desmosomes compared to controls. However, MDEOs maintained pore and leak pathway integrity, indicating that the duodenal epithelial barrier remained functionally preserved under tested conditions.

**Conclusion:**

This study provides evidence that the duodenal epithelium of MASH patients exhibits significant alterations in its nutrition-related and barrier functions. This study sheds light on the intricate dynamics of duodenal epithelial alterations in MASH, highlighting potential therapeutic avenues for restoring intestinal functions.

## Graphical abstract

**Figure:**
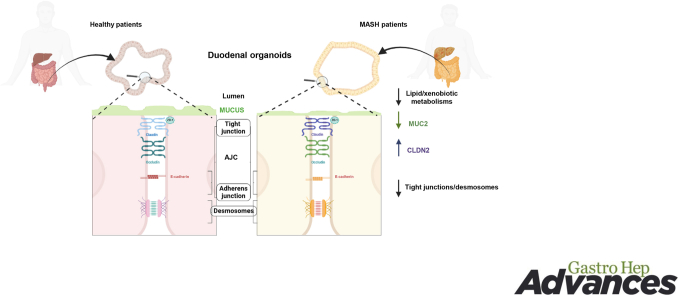

## Introduction

Metabolic dysfunction-associated steatotic liver disease (MASLD), formerly known as nonalcoholic fatty liver disease, is the leading cause of chronic liver diseases worldwide,^1^ while metabolic dysfunction-associated steatohepatitis (MASH) represents its advanced and progressive form.^2^, ^3^, ^4^

Although MASH constitutes the hepatic manifestation of the metabolic syndrome, its development and progression involve multiple signaling pathways arising from different body systems,^4^ primarily the adipose tissue and the gut. Furthermore, compelling evidence suggests that the gut-liver axis is intricately linked not only with the progression but also with MASH disease’s development. One of the key hallmarks is the disruption of the intestinal barrier integrity^5^ leading to the passage of bacteria and their metabolic products into the portal system, which ultimately worsens hepatic inflammation and drives metabolic alterations. More specifically, recent findings have shown that excessive fructose intake and high-fat diets (saturated fat) promote intestinal barrier’s disruption,^6^ increase villus length and therefore increase nutrient absorption and adiposity,^7^^,^^8^ and reduce mucous layer’s thickness,^9^ overall leading to systemic low-grade inflammation.^10^^,^^11^ These findings emphasize the putative role of the small intestinal epithelium in the development and disease progression of MASH, but the fact that the above findings result from studies on mouse models is a limitation in fully understanding the mechanisms involved. In humans, a growing number of studies evaluating bariatric surgery intervention points to a role of the most upper part of the small intestine, ie, the duodenum, in liver disease. Indeed, such procedures, including the commonly used Roux-en-Y Gastric Bypass, which bypasses part of the stomach and the duodenum, improve MASLD and promote substantial MASH disease resolution.^12^^,^^13^ Yet, the potential disruption of duodenal epithelium in MASH patients is still incompletely studied, thereby its investigation could be of great interest in deciphering potential therapeutic targets at the level of the intestinal mucosa. The breakthrough of intestinal organoid technology derived from individual patients has currently revolutionized translational research allowing the 3-dimensional growth of tissue in cell cultures derived from multipotent epithelial stem cells. The major advantage of 3-dimensional organoids (unlike human primary epithelial cell models) resides in their capacity to self-organize and renew, allowing them to faithfully recapitulate the features and functions of the intestinal villous-crypt unit by maintaining cell diversity, barrier function, genetic and epigenetic pattern that are highly similar to in vivo^14^ as already demonstrated in other gastrointestinal diseases such as inflammatory bowel disease.^15^, ^16^, ^17^ Consistently, few studies^18^^,^^19^ have already established liver organoids derived from MASH patients to further decipher the underlying molecular mechanisms at the level of the liver; however, so far, this model has not yet been used for studying gut epithelium.

Herein, we sought to further study the putative gut epithelium disruption by generating duodenal organoids derived from MASH patients and healthy subjects. To this end, we analyzed patient-derived organoid transcriptomes from these 2 groups to evaluate if specific intestinal pathways were differentially modulated. Our data reveal that the duodenal epithelium of MASH patients exhibits significant alterations in its absorptive and barrier functions, regardless of both luminal nutrient and microbial content and the surrounding subepithelial compartment.

## Results

### Generation of a Living Duodenal Organoid Biobank From Human MASH Patients

**Figure 1:**
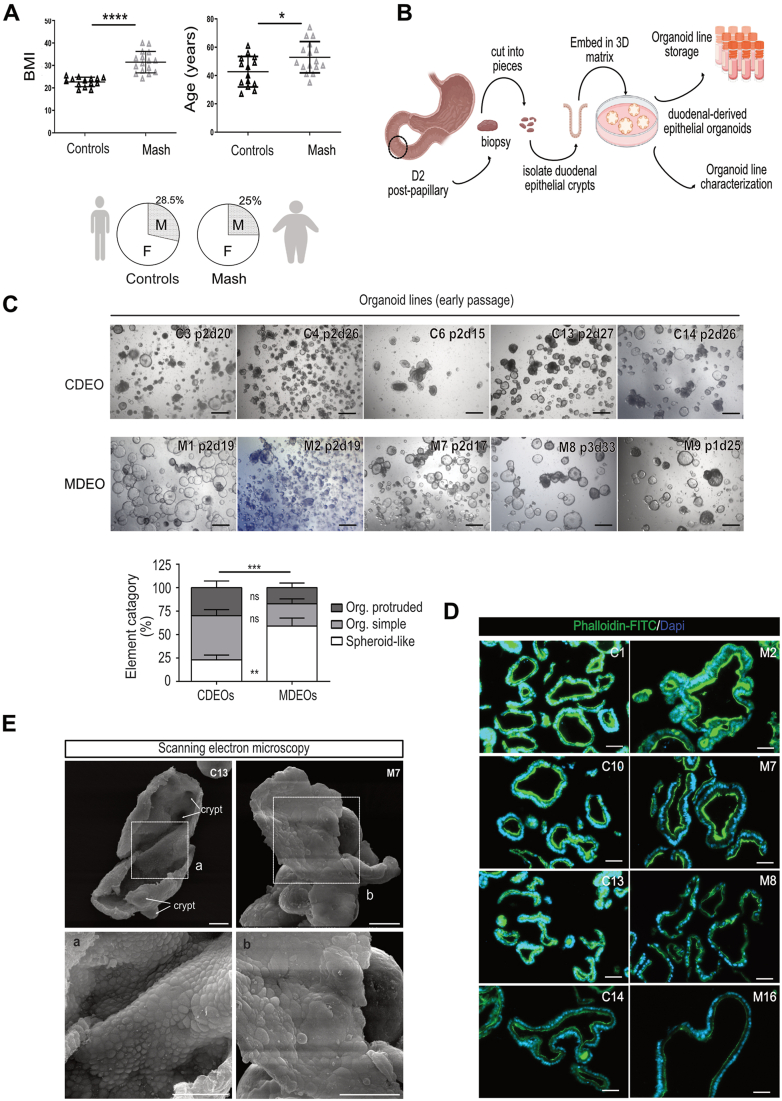
Generation of a living duodenal organoid biobank from human MASH patients. (A) Demographic characteristics of patients selected for this study. Data are represented as means ± standard deviation. Left panel: body mass index: unpaired *t* test: ∗∗∗∗*P* < .0001; Right panel: age: unpaired *t* test: ∗*P* < .05. Lower panel: Sex distribution. (B) Scheme of the workflow from patient to organoid line storage (created with Biorender.com). (C) Representative pictures of various CDEO and MDEO lines at the time of organoid line storage. Identity, passage, and day of culture are indicated for each organoid line. Scale bars: 500 μm. Lower panel: quantification of organoid complexity was determined for n = 4 CDEO and 5 MDEO lines at the time of organoid line storage (1–3 passages). Categories are expressed as the mean ± standard error of the mean. Two-way ANOVA (interaction ∗∗∗*P* < .001) followed by Sidak's multiple comparisons test: MDEO vs CDEO spheroids: ∗∗*P* < .001; MDEO vs CDEO: organoid simple or organoid protruded: not significant. (D) Representative pictures showing apical polarity in CDEO and MDEO lines using phalloidin-FITC. Nuclei were counterstained with 4′,6-diamidino-2-phenylindole. Scale bars: 50 μm. (E) Representative picture of scanning electron microscopy showing crypt domains (arrows) and luminal side in CDEOs and MDEOs with insets (a, b). Scale bars: 50 μm. ANOVA, analysis of variance; F, female; M, male; ns, not significant; Org, organoid.

To investigate the potential contribution of the duodenal epithelium to metabolic dysregulation associated with MASH, we generated an organoid biobank from human duodenum biopsies obtained from 16 biopsy-proven MASH patients (Figure 1A). Eligible MASH patients were adults with nonalcoholic fatty liver disease activity score ≥4, integrating the following parameters: steatosis, ballooning, and inflammation. The main inclusion and exclusion criteria are reported in the Materials and Methods section, while the type 2 diabetes status and total cholesterol levels of included patients are reported in Figure A1A. Duodenum biopsies obtained from 14 adult outpatients who underwent routine esophagogastroduodenoscopy in the setting of epigastric pain and gastroesophageal reflux disease were used as controls. In line with the clinical metabolic status of MASH patients, body mass index and age substantially differed between Control and MASH subjects. Despite the overrepresentation of females over males in patients, such sex bias was present in both clinical groups [28.5% vs 25% males in controls and MASH patients, respectively] (Figure 1A). Starting from an initial amount of 3 biopsies per patient collected in the second part of the duodenum (D2 postpapillary), we successfully generated 71% (10/14) control-derived duodenal epithelial organoid (thereby referred to as CDEOs) and 69% (11/16) MASH-derived duodenal epithelial organoid (thereby referred as MDEOs) lines. Organoids were amplified and stored as a biobank in the frame of 2 months following initial seeding (Figure 1B). Intra- and interindividual heterogeneity were observed for grown elements within organoid lines. However, MDEOs more frequently adopted cystic spheroid-like morphology as compared to CDEOs (Figure 1C). Of note, following freezing/thawing processes, 7/10 CDEO and 6/11 MDEO lines were efficiently replated beyond passage 5. Using the phalloidin-fluorescein isothiocyanate (FITC) compound on organoid sections, we first assessed cell polarity. Both CDEOs and MDEOs were made of lumen-oriented apically polarized cells (Figure 1D). Scanning electron microscopy further confirmed the presence of crypt-like domains in CDEOs as compared to less frequently protruded MDEOs (Figure 1E). On the luminal side, microvilli were present in organoids of both types of subjects (Figure 1E, insets). The cystic phenotype in MDEOs was maintained at passages 4/5 and beyond (Figure A1B).

Next, to determine whether MASH could be associated with any alterations in duodenal transcriptomic profiles, global transcriptomes of duodenal biopsies and duodenal-derived organoids isolated from control and MASH patients (n = 12 and n = 13, respectively) were obtained by bulk RNA sequencing. First, we controlled the tissue identity of biopsy and organoid samples by analyzing the expression of tissue-specific transcription factors. High levels of intestinal-specific CDX2 and CDX1, as well as PDX1 transcription factors were detected, in contrast to the foregut-related SOX2 factor, confirming the duodenal origin of samples (Figure A1C). We also excluded potential contamination of our organoid lines with CDX2-negative and Cadherin (CDH)-17-negative stem/progenitor cells from the submucosal Brunner’s glands^20^^,^^21^ (Figure A1D). For this purpose, immunofluorescence stainings were performed on patient biopsies and derived-organoid lines (Figure A1E and F). Epithelial cells forming CDEOs and MDEOs were detected as CDH17-positive and expressed the intestinal crypt stem cell marker Olfactomedin 4 (OLFM4) (Figure A1F). These data confirmed that organoid lines originated from duodenal crypts.

### MASH-Derived Organoids Exhibit Altered Tissue Homeostasis

**Figure 2:**
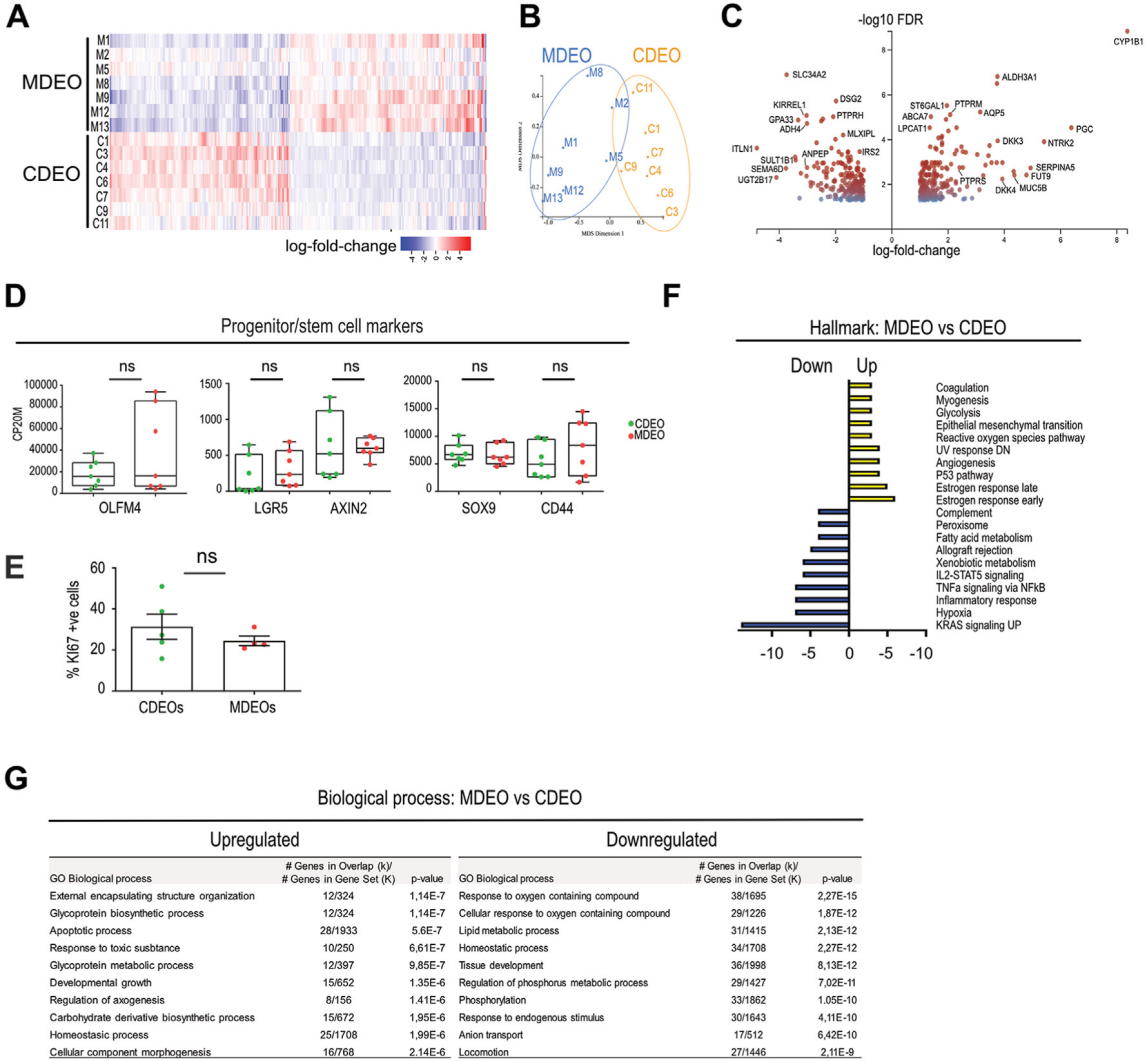
Characterization of tissue identity in patient biopsies and organoid lines. (A) Heatmap of DEGs in CDEOs and MDEOs (log 2-fold change). (B) Principal component analysis plot of control. MDS (C) and MASH (M)-derived organoid transcriptomes. Each dot refers to an individual subject. (C) Volcano plot showing transcriptome analysis of MDEOs vs CDEOs. Axes: x, log2-fold change; y, log10 of FDR. Some genes involved in metabolism, cell signaling, and cell adhesion are highlighted. (D) Expression levels of genes coding for progenitor/stem cell markers. CP20M: counts per kilobase of transcript per 20 million mapped reads. Each symbol corresponds to the value of an organoid line from an individual subject. Mann-Whitney test: not significant. (E) Cell proliferation index determined by the percentage of KI67-positive cells in organoids. Unpaired *t* test: not significant. Each symbol corresponds to the value of an organoid line from an individual subject. (F) GSEA-hall mark for upregulated and downregulated gene lists in MDEOs vs CDEOs. *P* value is indicated. (G) GSEA-biological processes for upregulated and downregulated gene lists in MDEOs vs CDEOs. DN, down-regulated; FDR, false discovery rate; GO, gene ontology; GSEA, gene set enrichment analysis; IL2-STAT5, interleukin 2-signal transducer and activator of transcription 5; MDS, multi dimensional scaling; NFkB, nuclear factor kappa-light-chain-enhancer of activated B cells; ns, not significant; UP, upregulated; UV, ultraviolet.

Next, we compared transcriptomic profiles of control and MASH-derived biopsies using the Degust software. However, no substantial differential gene expression could be observed between the groups when parameters were set to a False Discovery Rate of 0.05 and absolute log2-fold change of 1. Then, we compared duodenal epithelium in both cohorts by analyzing the transcriptome of their derived organoids (n = 7 CDEOs and 7 MDEOs) cultured under the same medium conditions for approximately 2 months after initial seeding. Using the same parameters as for biopsies’ analysis (ie, False Discovery Rate 0.05 and log2-fold change of 1), we identified 437 differentially expressed genes (DEGs), with 225 upregulated and 212 downregulated genes in MDEOs vs CDEOs (Figure 2A–C). Note that gene expression of Wnt signaling-associated intestinal stem cell markers (OLFM4, LGR5, AXIN2, SOX9, and CD44) did not significantly differ between MDEOs vs CDEOs (Figure 2D) and cell proliferation, evaluated by the percentage of KI67 positive (^+ve^) cells, was similar in both kinds of organoids (Figure 2E), indicating that stemness was preserved in the duodenal epithelium of MASH patients. However, analysis of the modulated “Hallmark” pathways revealed downregulation of the Kirsten rat sarcoma virus, hypoxia, inflammatory response, xenobiotic, and lipid metabolisms, with concomitant upregulation of the estrogen response early/late and p53 pathways as well as the reactive oxygen species pathway (including the G6PD, PRDX4, AKR1B1, ARMCX1, and LCN2 genes) in MDEOs vs CDEOs (Figures 2F, A2A). Investigation of the associated “Biological process” pathways revealed deregulation of tissue functionality (59 modulated genes from the “homeostatic process” dataset list). Transcriptome analysis indicated downregulation of “phosphorylation” processes (*P* value 1.05e-12) in MDEOs (Figure 2G). This was associated with increased expression levels of several receptor-type tyrosine-protein phosphatases (PTPRU, PTPRM, and PTPRS) reported to regulate the HIPPO/YAP and ERK signaling cascades^22^^,^^23^ (Figures 2G, A2B). Altogether, these in silico studies suggested dysregulated signal transduction in the duodenal epithelium of MASH patients as compared to healthy subjects.

### Modified Nutrient Absorption Potential in Duodenal-Derived Organoids of MASH Patients

**Figure 3:**
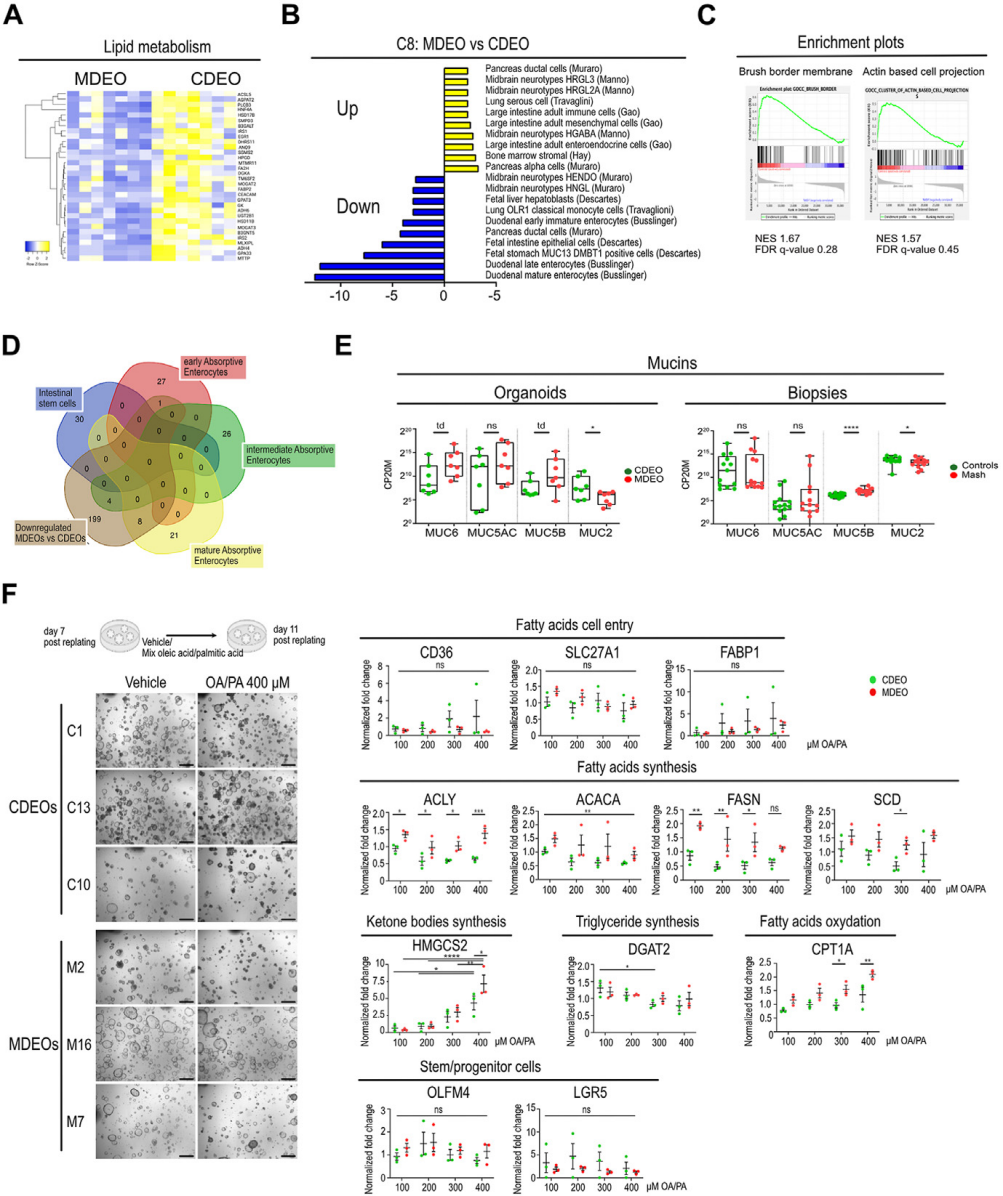
Transcriptome analysis of MASH-derived organoids reveals altered homeostasis. (A) Heatmap showing differentially modulated genes involved in lipid metabolism. (B) GSEA-C8 “cell type signature” for upregulated and downregulated gene lists in MDEOs vs CDEOs. *P* value is indicated. (C) GSEA showing de-enrichment of the “Brush border membrane” and “Actin-based cell projection” dataset in MDEOs vs CDEOs. (D) Venn diagram showing that genes expressed by mature absorptive enterocytes, as defined by Gomez-Martinez et al (2022), are downregulated in MDEOs vs CDEOs. (E) Expression levels of genes coding for various mucins. CP20M: counts per kilobase of transcript per 20 million mapped reads. Each symbol corresponds to the value of an organoid line or biopsy obtained from an individual subject. Mann-Whitney test: ∗∗∗∗*P* < .0001; ∗*P* < .05; td: *P* < .1. (F) Organoid challenge with a mix of OA and PA for 4 days starting at 7 days postreplating. Left panel: Representative pictures of several organoid lines challenged with OA/PA (400 μM) or the vehicle. Scale bars: 500 μm. Right panel: Gene expression analysis by quantitative reverse transcription polymerase chain reaction of the indicated genes involved in lipid metabolism. Each symbol corresponds to a given organoid line. Two-way ANOVA tests with Sidak’s multiple comparisons. ∗∗∗∗*P* < .0001; ∗∗∗*P* < .001; ∗∗*P* < .01; ∗*P* < .05. ANOVA, analysis of variance; FDR, false discovery rate; GSEA, gene set enrichment analysis; NES, normalized enrichment score; ns, not significant; OA, oleic acid; td, tendency.

Strong downregulation of “response to oxygen-containing compound” processes (*P* value 2.27e-15) was detected in MDEOs vs CDEOs (Figure 2G). Expression levels of genes associated with “lipid metabolic processes” (FABP2, DGKA, ACSL5, MOGAT3, SMPD3, GPA33, etc.) were reduced in MDEOs (Figure 3A). Several genes involved in carbohydrate metabolism were also downregulated, such as SGK1 regulating intestinal glucose absorption, the key regulator of lipid/carbohydrate metabolisms MLXIPL/CHREBP gene, as well as the pivotal insulin-associated signaling molecule insulin receptor substrate 2 (Figures 2C, A2A). Moreover, glucose transport mediated by solute carrier (SLC) protein-coding genes was altered, with downregulation of SLC2A2/GLUT2 and SLC5A1/GLUT1 and upregulation of the SLC2A10/GLUT10 (Figure A3A). In addition, the expression of SLCs involved in amino acid, nucleoside, and ionic transport was also deregulated in MDEOs (Figure A3A). Analysis of DEGs using the C8 “cell type signature” gene set indicated downregulation of immature, mature, and late enterocytes-associated markers in MDEOs vs CDEOs (Figure 3B). Coherent with these results, we found de-enrichment of the “Brush Border membrane” and “Actin filament bundle” gene datasets in MDEOs vs CDEOs (Figure 3C). Moreover, 8 out of 29 genes (namely *SGK1*, *CDHR5*, *ALDOB*, *SLC5A1*, *FABP2*, *SLC13A2*, *ANPEP*, and *SI*) of the human absorptive mature lineage list established by Gomez-Martinez et al^24^ were identified within the list of 212 downregulated genes in MDEOs vs CDEOs (Figure 3D). Regarding secretory lineages, we noticed a slight upregulation of gastric mucins (MUC5B and MUC6) with a concomitant decrease in the goblet marker MUC2 in MASH-derived organoids and biopsies (Figures 3E, A3B). Nevertheless, this was not accompanied by a manifest reduction in goblet cell density on biopsies (Figure A3C). Altogether, these data suggested either biased commitment of stem cells toward the secretory vs absorptive lineages or reduced absorptive maturation of committed progenitors in MDEOs as compared to CDEOs. Next, having found evidence for altered digestive functions, we next compared the lipid metabolic potential of MDEOs and CDEOs. Organoids were stimulated 7 days postreplating with a mixture of oleic acid and palmitic acid (PA), 2 major fatty acids of the diet, for a further 4 days in culture. Globally, treatment promoted the morphological conversion of spheroids into organoids, although MDEO lines exhibited heterogeneity (Figure 3F, left panel). Expression of genes involved in fatty acid cell entry or stem/progenitor markers did not appear significantly modulated by oleic acid/PA challenge (Figure 3F, right panel). However, lipid challenge reduced the expression of genes associated with fatty acid and triglyceride synthesis in a dose-dependent manner, and higher expression levels of ACLY, ACACA, and FASN were detected in MDEOs vs CDEOs. Conversely, the expression of CPT1A and HMGCS2 genes, involved in fatty acid beta-oxidation and ketone bodies synthesis, respectively, was stimulated by increasing concentrations of the mixture. MDEOs exhibited higher fatty acid oxidation potential as compared to CDEOs (Figure 3F, right panel). Taken together, these results indicated that MDEOs maintain their lipid metabolic capacity despite suggested lineage commitment bias, potentially through lipid oxidation mediated by stem/progenitor cells.

### Misexpression of Cell Adhesion Components in Duodenal-Derived Organoids of MASH Patients

**Figure 4:**
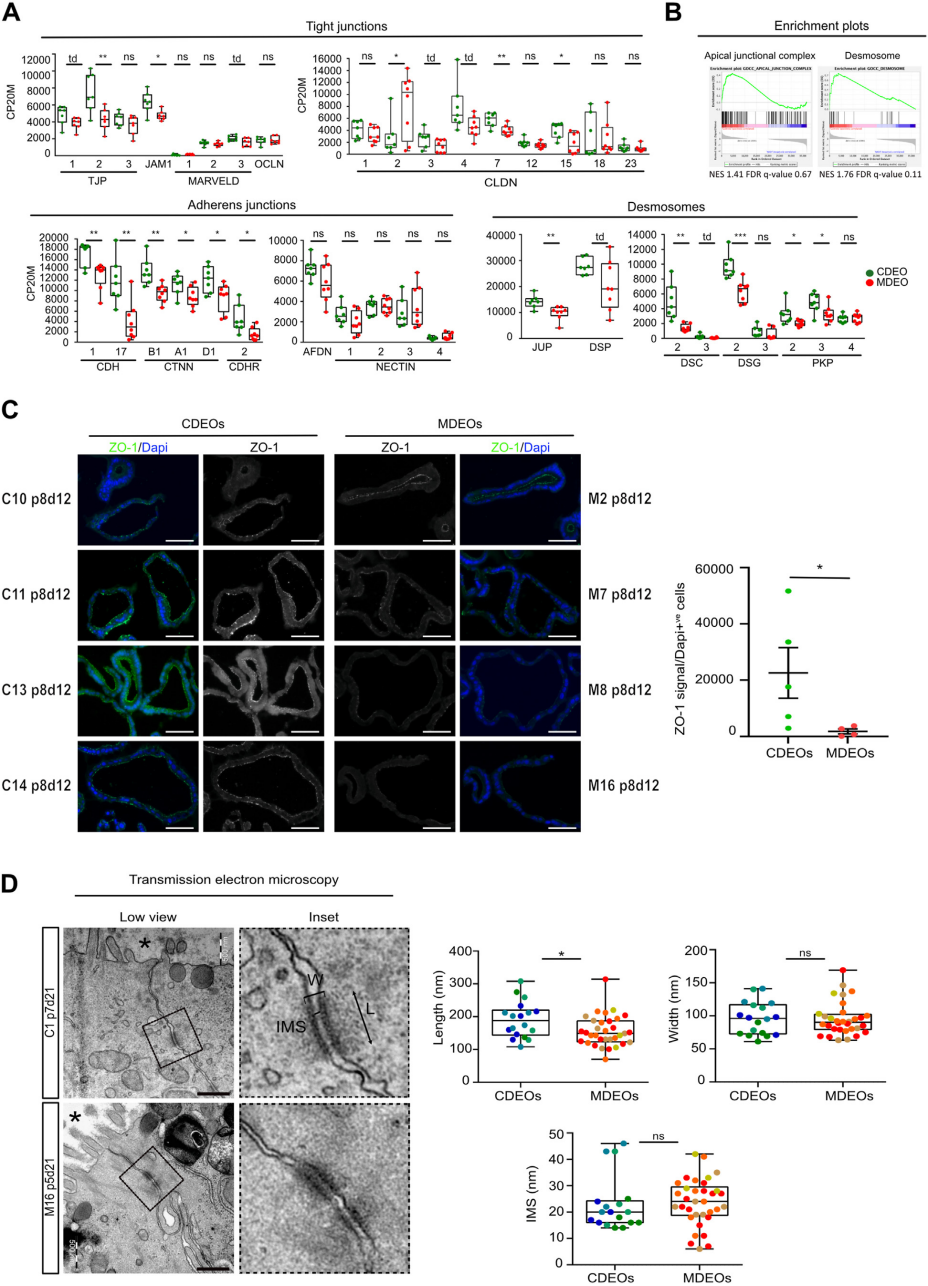
Transcriptome analysis of MASH-derived organoids reveals dysregulated expression of cell junction components. (A) Expression levels of genes coding for components of tight and adherens junctions and desmosomes. CP20M: counts per kilobase of transcript per 20 million mapped reads. Each symbol corresponds to the value of an organoid line obtained from an individual patient. Mann-Whitney test: ∗∗∗*P* < .001; ∗∗*P* < .01; ∗*P* < .05; td: *P* < .1. (B) GSEA showing de-enrichment of the “Apical junctional complex” and “Desmosome” datasets in MDEOs vs CDEOs. (C) Representative pictures of immunofluorescence showing discrete expression of ZO-1 in tight junctions in organoids. Nuclei counterstained with 4′,6-diamidino-2-phenylindole. Scale bars: 50 μm. Right panel: quantification of ZO-1 expression levels relative to the total number of cells. Each symbol refers to an individual organoid line (n = 5 CDEOs, n = 4 MDEOs). A mean of 267 cells were analyzed per organoid line. Mann-Whitney test: ∗*P* < .05. (D) Representative pictures of transmission electron microscopy showing altered desmosome architecture in MDEOs. Asterisks indicate the luminal side of organoids. Insets show higher magnification of desmosomes. Scale bars: 500 nm. Quantification of the first most apical desmosome in MDEOs and CDEOs. Each symbol represents an individual desmosome and colors identify individual CDEO (n = 4) or MDEO (n = 4) lines. Unpaired *t* test: ∗*P* < .05, ns: not significant. FDR, false discovery rate; GSEA, gene set enrichment analysis; IMS, intermembrane space; L, length; NES, normalized enrichment score; ns, not significant; W, width.

Since mouse MASH model studies have reported disruption of intestinal epithelial barrier integrity,^5^ we analyzed expression levels of components of the apical junctional complexes. Expression of several tight junction components (TJP1/ZO1, TJP2/ZO2, and JAM1/F11R) was found downregulated in MDEOs (Figures 4A and B, A4A). Claudins, the other structural and functional components of tight junctions, also exhibited altered expression profiles. Nine out of 24 genes encoding members of this family of proteins were expressed at significant levels (CLDN1, 2, 3, 4, 7, 12, 15, 18, 23) in organoids. CLDN7, CLDN15, and CLDN4 (to a lesser extent) were downregulated in MDEOs (Figure 4A). Adherens junction components, which bring mechanical strength at cell-cell adhesion sites and contribute to cell polarization, were also dysregulated in MDEOs vs CDEOs. Indeed, cadherins (CDH1, CDH17, and CDHR2) and catenins (CTNNB1, CTNNA1, and CTNND1), but not afadin or nectins, were significantly downregulated in MDEOs vs CDEOs (Figure 4A). Desmosome components (junction plakoglobin, desmoglein-2, desmocollin-2, plakophilin 2, and plakophilin 3 ) were also found to be downregulated in MDEOs as compared to CDEOs (Figure 4A). In line with transcriptomic data, the protein TJP1/ZO-1 was detected regularly spaced with punctuate apical staining in CDEOs but much less clearly visualized in MDEOs at any of the Z-stack focal planes analyzed (Figure 4C). Moreover, despite similar expression levels at the transcriptional level, the protein occludin, another tight junction component, localized more diffusely in MDEOs than in CDEOs (Figures 4A and A4B). Immunofluorescence studies using anti-DSC-2 antibodies, performed to visualize desmosomes at the intercellular junctions of the epithelium, suggested lower signal in MDEOs vs CDEOs (Figure A4C). Since the loss of desmocollin 2 in the mouse intestine results in desmosome ultrastructure alterations,^25^ we further analyzed the first most apical desmosome detected at the cell-cell junction by transmission electron microscopy (Figure 4D). This revealed shorter desmosomes in MDEOs as compared to CDEOs (Figure 4D). Altogether, these data indicated substantial intrinsic alterations of the cell-cell adhesion potential in MASH-derived organoids as compared to controls.

### The Intestinal Epithelial Barrier is Functionally Preserved in MDEOs

**Figure 5:**
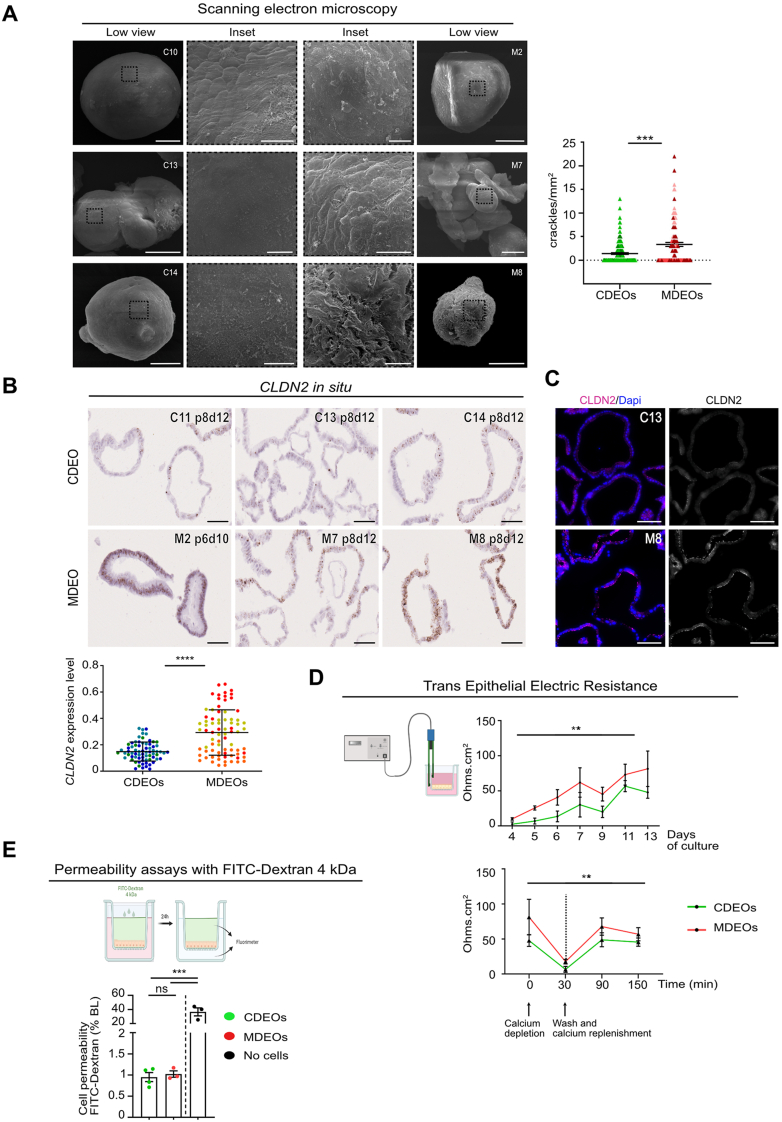
Preserved cell permeability of MASH duodenal-derived organoids. (A) Representative pictures of scanning electron microscopy (inset: higher magnification) showing crackled areas at the BL surface of MDEOs. Scale bars: 50 μm, insets: 10 μm. Right panel: quantification of the number of crackles per mm^2^ of organoid surface performed on n = 4 CDEOs and n = 3 MDEOs (35–45 pictures analyzed per organoid line). Mann-Whitney test: ∗∗∗*P* < .001. (B) Expression of the leaky gut-associated *CLDN2* gene detected in various CDEOs and MDEOs by RNAscope. Scale bars: 50 μm. Right panel: quantification of CLDN2 expression levels performed on n = 3 CDEOs and n = 3 MDEOs (each line is identified with a different color) with a mean of 25 elements analyzed per organoid line. Data are expressed as the mean ± standard deviation. Mann-Whitney test: ∗∗∗∗*P* < .0001. (C) Representative pictures of immunofluorescence showing reinforced apical expression of CLDN2 in MDEOS. Nuclei counterstained with 4′,6-diamidino-2-phenylindole. Scale bars: 50 μm. (D) TEER measurement performed on organoid lines grown on 2D cell inserts over time (left panel) or during a calcium depletion challenge (right panel) on n = 4 CDEOs and n = 3 MDEOs. Left panel: Two-way ANOVA (time ∗∗∗∗*P* < .0001, cohort ∗∗*P* < .01) followed by Tukey's multiple comparisons test: MDEOs vs CDEOs d13: ∗*P* < .05; CDEOs d4/d5 vs d11: ∗∗*P* < .01, d4/d5 vs d13: ∗*P* < .05, d6 vs d13: ∗*P* < .05; MDEOs d4 vs d7: ∗*P* < .05, d4 vs d11: ∗∗*P* < .01, d4 vs d13 ∗∗∗*P* < .001, d5 vs d11 ∗*P* < .05, d5 vs d13 ∗∗*P* < .01. Right panel: Two-way ANOVA (time ∗∗*P* < .01, cohort ∗∗*P* < .01) followed by Tukey's multiple comparisons test: MDEOs vs CDEOs: ns, not significant; CDEOs at 30 minutes vs 90 minutes and CDEOs at 30 minutes vs 150 minutes: ∗*P* < .05. (E) FITC-dextran 4 kDa permeability assays performed on organoid lines grown on 2D cell inserts. Permeability is expressed as the percentage of tracer in the BL compartment after 24 hours. One-way ANOVA test with tukey’s multiple comparisons. ∗∗∗∗*P* < .0001. CDEOs vs no cells: ∗∗∗*P* < .001; MDEOs vs no cells: ∗∗∗*P* < .001; MDEOs vs CDEOs: not significant. ns, not significant.

To study the potential functional consequences of cell-cell adhesion modifications in MASH organoids, the ultrastructure of the basolateral (BL) surface of organoids was analyzed by scanning electron microscopy. As shown in Figure 5A, meanwhile, the BL surface was globally smooth for the various CDEOs; MDEOs exhibited a higher density of crackles, this feature revealing looser cell-cell interactions in disease-related organoids (Figure 5A). Moreover, transcriptome analysis revealed that the CLDN2 member, involved in tight junction formation, was upregulated in MDEOs vs CDEO (Figure 4A). To confirm these data, we quantified its expression levels by RNAscope and found globally increased levels in MDEOs, despite substantial heterogeneity among organoid lines and detected apical reinforcement of CLDN2 in MDEOs as compared to CDEOs (Figure 5B and C). Paracellular permeability of the epithelial barrier depends both on the pore pathway, a high-capacity size and charge selective for small ions and solutes, and the leak pathway involved in the transcellular movement of larger solutes.^26^ Since CLDN2 is recognized as an essential tight junction component in the pore pathway, we assessed epithelial barrier integrity in control and MASH-derived cultures.^27^ For this purpose, the transepithelial electric resistance (TEER) of organoid lines, used as a surrogate marker of the pore pathway, was analyzed by culturing cells in 2D on transwells coated with Matrigel (Corning). Except for the MDEO 8 lines, unable to form a monolayer, the establishment of a complete monolayer was monitored over time by brightfield observations; this was further confirmed by phalloidin-FITC staining (Figure A5A and B). As expected, this correlated with progressive TEER increase in both kinds of cohorts (Figure 5D, upper panel). Cell monolayers were made of stem/progenitor OLFM4^+ve^, differentiated absorptive VILLIN^+ve^/and secretory Goblet UEA-I^+ve^ and LYZ^+ve^ Paneth cells (Figure A5C). Next, we investigated the tight and adherens junction reassembly potential following a calcium depletion challenge performed on fully grown monolayers on day 13 postseeding. As expected, such treatment led to a sharp reduction in TEER within 30 minutes, whereas calcium replenishment was associated with TEER restoration within 2 hours (Figure 5D, lower panel). However, these calcium challenges induced similar responses regardless of the organoid line origin (Figure 5D, lower panel). We also studied the macromolecular paracellular permeability leak pathway using the FITC-dextran 4 kDa as a tracer molecule to explore the noncharged selective pathway. Cell permeability, calculated as the percentage of BL tracer detected after 24 hours, did not substantially differ between the 2 groups (Figure 5E). Together, these experiments indicated that despite modified cell adhesion properties of MDEOs vs CDEOs, permeability to ions, small molecules, and macromolecules was globally preserved in MASH-derived epithelium under the tested culture conditions.

## Discussion

In this study, we provide new evidence that the duodenal epithelium of MASH patients exhibits significant alterations in its nutrition and barrier functions. This was observed in a culture model that isolated the epithelium to assess its intrinsic functional changes, irrespective of luminal nutrient and microbial contents, as well as the surrounding subepithelial compartment.

We used the organoid approach to generate a biobank of duodenal organoids obtained from control subjects and MASH patients. The success rate of this study, involving a limited number of individuals, was found to be similar in both cohorts, ie, approximately 70%, indicating that MASH-derived stem cells had maintained their stemness capacity ex vivo. Consistent with previous reports on human liver organoid models for MASLD and MASH,^18^^,^^19^^,^^28^^,^^29^ our study revealed both inter- and intraorganoid line heterogeneity in terms of morphology and gene expression within both types of organoids. Multiple differences in the origin of the duodenal samples obtained from individuals with distinct ages, clinical presentations, and dietary habits likely contribute to such variability. Nevertheless, despite this heterogeneity, a consistent phenotype was observed in MASH-derived organoids over passages. Of note is that such differences were not detectable on biopsies at the transcriptomic level (except for the highly epithelial-specific mucin-encoded genes), which further highlights the potential of organoid technology for clinical studies. Persistence of the cystic morphology in MDEOs, which did not appear to correlate with differential cell proliferation rate in stem/progenitor cells, might be explained in part by differential expression of ionic and water flux SLCs and aquaporin transporters, as revealed by bulk RNA sequencing.

Transcriptome profiling of MASH and control-derived organoids revealed global dysregulation of tissue homeostasis associated with reduced response to organic compounds in disease-associated organoids, involving downregulation of lipid and xenobiotic metabolisms. These findings were suggestive of reduced absorptive cell lineage terminal differentiation; meanwhile, secretory markers normally expressed in the anterior stomach (MUC5AC, MUC5B, MUC6, and PGC), as well as several secretory or neuroendocrine markers (such as SCG5, PROX1, RETREG1, and MAP1), were found upregulated in MASH organoids. Even though we failed to morphologically identify these neuroendocrine cells, our data are collectively suggestive of biased stem cell/progenitor lineage commitment in MASH organoids. Such observations are in line with recent single-cell RNAseq studies performed on mouse models showing the high degree of plasticity of the intestinal epithelium, able to modulate gene expression in response to acute or chronic nutrient challenges.^30^, ^31^, ^32^ Indeed, an acute high-fat diet leads to the appearance of new trajectories for secretory and enterocyte lineages associated with an increase in the stem cell/progenitor pools.^30^ In a high-fat/high-sugar diet model, single-cell RNA sequencing studies have revealed a transcriptomic shift occurring in the small intestinal epithelium toward a more anterior cell type identity.^31^ In a human pathological context, increased expression of gastric mucins has been reported in the ulcer margins of ileal mucosa in Crohn’s disease.^33^ Accordingly, our data collectively suggest that MDEOs exhibit altered lineage differentiation compared to CDEOs when cultured under the same conditions. In the future, complementary single-cell RNA sequencing studies should help to fully characterize the altered stem/progenitor trajectories in MASH-derived organoids.

MASH is associated with the accumulation of lipids in hepatocytes, leading to steatosis and progressive development of inflammation, which may result in the evolution of liver fibrosis.^4^ Previous ex vivo studies performed on human organoids generated from either induced pluripotent stem cells or direct liver bipotent ductal cells have reported impaired lipid oxidative metabolism leading to lipid droplet accumulation in MASH disease-related organoids or upon lipid challenge, as well as higher sensitivity to apoptosis following PA stimulation.^18^^,^^19^^,^^28^^,^^29^^,^^34^ In the present work, having provided transcriptomic evidence for dysregulated lipid metabolism in MASH duodenal epithelium, we explored their lipid metabolic potential upon free fatty acid stimulation. In line with mouse studies, fatty acids induced dose-dependent expression of CPT1A, regulating fatty acid access to the mitochondrial matrix for β-oxidation and the HMGCS2 enzyme promoting mitochondrial ketone body formation and reported necessary to maintain intestinal stemness.^32^^,^^35^^,^^36^ However, we also found higher expression levels of genes involved in fatty acid biosynthesis in MASH vs control organoids. Overall, these experiments demonstrated the capacity of MASH organoids to metabolize fatty acids in response to a challenge despite an observed global reduction in lipid metabolic processes. Future experiments meant to visualize the accumulation of lipid droplets in duodenal organoids would help to determine whether diseased organoids do exhibit preponderant lipid anabolic rather than catabolic processes as compared to controls.

Mouse models of MASH have provided evidence that liver inflammation is favored by gut-derived bacteria and bacterial metabolites following the initial disruption of intestinal barrier integrity.^5^ In humans, previous studies have shown less clear conclusions, but a recent meta-analysis review has correlated increased intestinal permeability in MASLD patients with the degree of liver steatosis.^37^ Moreover, a lifestyle that promoted weight loss successfully reversed the increased intestinal permeability and the extent of liver steatosis in obese patients.^38^ Various interdependent players, including the epithelial barrier itself, microbiota, immune, stromal, and enteric neurons, contribute to maintaining intestinal barrier integrity.^39^ Interestingly, the present study, focused on the sole epithelial player, has uncovered dysregulated expression of important components of cell-cell interaction, including the tight junction component CLDN2, one of the few members of this family of proteins, with pore-forming cation-selective channel properties.^26^ Of relevance, expression of this claudin is reported to be upregulated in several pathophysiological conditions such as inflammatory bowel diseases, celiac disease, or irritable bowel disease.^26^^,^^27^ Nevertheless, it has recently been proposed that this claudin, detected in regenerative crypts of human inflammatory bowel diseases, would promote mucosal healing in a mouse experimental colitis model.^40^ Thus, the upregulation of CLDN2 in MASH vs control organoids observed in the present study might reflect a response to endogenous cellular stress occurring under the defined cultured conditions; this would be coherent with the observed concomitant upregulation of a redox response in diseased organoids. However, another not mutually exclusive interpretation for CLDN2 upregulation in MASH organoids is also plausible. Indeed, differential expression of claudins has been reported along the crypt-villus axis; meanwhile, claudin 7 is highly expressed in differentiated cells of the villi, and the highest levels of claudin 2 are detected in crypt domains.^41^ Therefore, the increased expression of CLDN2 in MASH organoids could be explained by relative amplification of the stem/progenitor pool as compared to control organoids. Certainly, additional studies are needed to fully decipher the function of this tight junction component in metabolic- and inflammation-related pathophysiological conditions. In addition to tight junction components, we also observed desmosome alterations and brittle BL cellular sides in MASH organoids by electron microscopy, suggesting reduced epithelial barrier potential. However, similar TEER and cell permeability to macromolecules were measured in MASH and control epithelia. In agreement with these findings, no significant differences were reported between severely obese and control subjects in intestinal permeability, despite evidence of morphologically altered tight junctions in patient tissues, unless a lipid challenge was performed.^42^ Therefore, it is likely that the intrinsic transcriptomic and morphological alterations related to cell-cell adhesion detected in MDEOs vs CDEOs are not by themselves the unique drivers of the suggested in vivo gut permeability in MASH patients, but they certainly contribute to initiating the process. In addition, it is expected that the observed reduced production of secreted and glycocalyx-associated membrane-bound mucins can also further dampen epithelial barrier integrity and potentially modify the microbiota. Finally, the subtle default of the BL epithelial barrier observed by scanning electron microscopy could also favor the access of stromal and immune cells to luminal content and thereby stimulate local inflammation. Further studies combining co-cultures of organoids with subepithelial cell types should help in the future to test this hypothesis.

In summary, despite the small number of samples involved in this study aimed at investigating the intrinsic characteristics of duodenal epithelia from MASH patients, we have revealed unique persistent alterations of disease-associated organoids. Since one of the most efficient treatments for MASH is lifestyle intervention, we anticipate that the duodenal organoid model could help test new therapeutic agents in the future to fully restore intestinal function.

## Materials and Methods

### Human Duodenal Tissues

Human small intestine (duodenum) samples were obtained from biopsy-proven MASH patients who were included in a pilot study assessing the effect of an endoscopic duodenal mucosal resurfacing on liver histology outcome.^43^ Control duodenum samples were obtained from outpatients who underwent routine esophagogastroduodenoscopy in the setting of epigastric pain and gastroesophageal reflux disease. Inclusion and exclusion criteria are reported in Supplementary Methods. All patients provided written informed consent. Clinical data for MASH patients are reported in Figure A1A.

### Crypt Isolation and Human Duodenal Organoid Culture

Crypt isolation and organoid culture from human duodenal samples (3 biopsies) were performed based on the reported protocol,^17^^,^^44^ which are detailed in Supplementary Methods. Images were acquired with a Moticam Pro camera connected to a Motic AE31 microscope. Quantification of organoid morphology was performed in single-blinded with pictures acquired during culture between passages 1–6 on n = 8 CDEO and 7 MDEO lines. A mean number of 135 elements were analyzed per organoid line (with a minimum of 36 elements analyzed for the CDEO 6 line).

### Tissue Processing, Immunohistochemical Analysis, and In Situ Hybridization

Biopsies were fixed in paraformaldehyde 4% for 24 hours at +4 °C and then processed for paraffin embedding (pathology laboratory, Institut Jules Bordet, Hôpital Universitaire de Bruxelles). Samples were sectioned at 5 μm using the Sakura Tissue-Tek AutoSection microtome. Organoid culture samples were harvested, prefixed in 10% formalin solution during 30 minutes at room temperature, and subsequently sedimented through 20% and 30% sucrose at 4 °C before optimal cutting temperature compound embedding (Table A1-reagents). Optimal cutting temperature compound samples were sectioned at 6 μm sections using a CM3050 cryostat (Leica Microsystems GmbH). Staining, imaging, and quantification procedures are detailed in Supplementary Methods.

### Gene Expression Analyses

RNA extraction, RNA sequencing, and Gene Set Enrichment Analysis were performed as detailed in Supplementary Methods. DEGs were identified with the EdgeR method (Degust) and further analyzed using Gene Set Enrichment Analysis MolSig (Broad Institute).^45^ Heatmaps were generated using Heatmapper.^46^

### Transepithelial Electrical Resistance Measurements and Paracellular Permeability Assay

To study cell permeability, organoid-generating cells were grown onto transwell inserts precoated overnight with 1% Matrigel (500,000 cells seeded/well of p24 well plates, diameter: 6.5 mm, pore: 0.4 μm). BL and apical chambers were filled with 600 μl and 200 μl of complete culture medium, respectively. The medium was changed every other day. Transepithelial electrical resistance was followed over time using an epithelial volt-ohmmeter (epithelial volt-ohmmeter device with STX4 electrodes). Experiments, further described in Supplementary Methods, were repeated at least twice for each organoid line; each measure was performed in triplicate.

### Statistical Analysis

Statistical analyses were performed with GraphPad Prism 10. All experimental data are expressed as mean ± standard error of the mean unless indicated in Figure legends. The significance of differences between groups was determined by appropriate parametric or nonparametric tests as described in Figure legends.

## Supplementary Materials

Supporting Documents
