# Supplementary material for: Duodenal Organoids From Metabolic Dysfunction-Associated Steatohepatitis Patients Exhibit Absorptive and Barrier Alterations
Source: Gastro Hep Adv. 2024 Dec 12;4(4):100599. doi: 10.1016/j.gastha.2024.100599 (PMC11849614; doi:10.1016/j.gastha.2024.100599)
Supplement: Supporting Documents [file mmc1.docx]

**SUPPORTING DOCUMENTS**

***SUPPLEMENTARY METHODS***

***Human duodenal tissues, exclusion and inclusion criteria***

Eligible MASH patients were adults (aged ≥ 18 years) with histological evidence of definite steatohepatitis (per central expert pathologist reading of a liver biopsy obtained ≤ 6 months prior to the procedure) with an MAFLD activity score (NAS) of at least 4, including at least one point each for steatosis, lobular inflammation, hepatocellular ballooning; and fibrosis stage, per Brunt stage, of F0 to F3. Main exclusion criteria were as follows: evidence of another cause of liver disease; history of sustained alcohol ingestion defined as daily alcohol consumption > 30 g/day for males and > 20 g/day for females; previous gastrointestinal surgery such as Billroth 2, Roux-en-Y gastric bypass, or other similar procedures or conditions; for subjects with type 2 diabetes, no current use of insulin or GLP-1 analogs; and for type 1 diabetes, probable insulin production failure defined as fasting C-peptide serum < 1 ng/mL. Control duodenal tissues were obtained from adults (aged ≥ 18 years) with body mass index < 25 with the following exclusion criteria: patients with type 2 diabetes, use of probiotics or antibiotics in the past 3 months.

***Crypt isolation and human duodenal organoid culture***

Biopsies were collected in the chelating buffer (CB) [constituted by cold sterile phosphate-buffered saline (PBS) supplemented with 2% sorbitol, 1% sucrose, 1% bovine serum albumin, gentamicin, and amphotericin B] (see Table 1-reagents). Briefly, villi were removed using fine scissors and the rest of the biopsy was further minced into 1 mm3 pieces and dissociated with 2 mM EDTA for 30 minutes at + 4°C with gentle rocking (90 rpm) to release crypts. Then, samples were centrifuged at 150 g for 5 minutes and resuspended in 5 mL of CB. Mechanical dissociation was carried out to release crypts by performing 40 times up and down pipetting with 5 mL pipette coated with fetal bovine serum. This fraction was next passed through a 100 µm-cell strainer. Filtered crypts were pelleted at 150 g for 5 minutes and suspended in Matrigel Growth factor reduced, Phenol-red free, LDEV-free onto 12-well plates as 100µl droplets. After Matrigel polymerization, samples were supplied with 700 µl culture medium and incubated at 37°C in a 5% CO2 cell culture incubator (Binder C150). The culture medium consisted of Advanced Dulbecco’s Modified Eagle’s medium supplemented with 5 U/ml penicillin/streptomycin cocktail, 40 µg/ml gentamycin, 2.5 µg/ml amphotericin B, 10 mM HEPES, 2 mM GlutaMAX, 1 mM N-acetyl cysteine, N2 supplement 1x, B-27 supplement without vitamin A 1x, 1 µg/ml human R Spondin-1, 100 ng/ml human Noggin, 50 ng/ml human recombinant epidermal growth factor, 10 nM Gastrin, 500 nM A83-01, 10 µM SB202190 and 10 mM of Nicotinamide (all indicated at the final concentrations, see Table 1-reagents). Culture medium was supplemented with 50% Wnt3A-conditioned medium (cell line Wnt-3A, ATCC, CRL-2647) containing 10% Fetal Bovine Serum, 1% Penicillin/Streptomycin, 10 mM HEPES, 2 mM GlutaMAX, as described (41). At initial seeding, 3,6 µM CHIR99021 and 2,5 µM thiazovivin were added to the medium. Culture medium was changed every two to three days. Organoid cultures were passaged every 15 to 20 days at a ratio of 1:5 using enzymatic dissociation with TrypLE express. Upon replating, culture medium was supplemented with 10 µM Y-27632 for the initial 2 days. For free fatty acid challenges, organoids were incubated 7 days after replating for 4 days with a free fatty acid mixture (stock solutions: oleic acid: 300 mM, palmitic acid: 250 mM final; 200 mM BSA) in complete culture medium.

***Tissue processing, immunohistochemical analysis and in situ hybridization***

For immunofluorescence or immunochemistry, antigen retrieval was performed by microwaving sections in 10 mM sodium citrate buffer, pH 6.0 before sample permeabilization (0.1 % Triton X-100 in DPBS). Nonspecific antibody binding was blocked for 1 hour at RT with blocking buffer (permeabilization buffer containing 5 % horse serum, 1 % BSA) before primary antibodies incubation overnight at + 4°C in blocking buffer. Sections were rinsed three times in PBS and incubated with secondary antibodies during 1 hour at RT. The ABC kits and substrate Kits (both from Vector Labs) were used for immunohistochemistry revelation. For fluorescence stainings, filamentous actin, lipids and nuclei were stained with phalloidin-FITC and DAPI, respectively. Slides were mounted with Fluorsave reagent. Antibodies and staining reagents are listed in Table 1-reagents. *In situ* hybridization experiments were performed on 6 µm-OCT embedded sections with the RNAscope kit (ACD-Biotechne) according to manufacturer instructions using the CLDN2 probe. Imaging was performed on a Zeiss Axio Imager M2 fluorescence microscope with a Zeiss Axiocam 503 mono camera and a Zeiss microscope Axio Observer 7 with axiocam MRN camera for immunofluorescence microscopy using Zen Pro software (Zeiss). Brightness, contrast, and picture size were adjusted using Zen lite software and fixed with a same setting for all pictures (Zeiss). Imaging for samples stained with antibodies or RNAscope probe by immunohistochemistry was performed with a Nanozoomer digital scanner (Hamamatsu). Quantification of CLDN2 expression levels was done with Q path 5.0 software by measuring the surface area (in µm^2^) of the probe signal over the surface area of the epithelial surface (in µm^2^) given by the hematoxylin signal. Results are presented as the ratio of CLDN2 signal/epithelium.

***Transmission electron microscopy (TEM) and scanning electron microscopy (SEM)***

Organoids collected between day 16 to day 21 after replating (between passage 5 and 12) were washed with PBS and fixed with ice-cold glutaraldehyde 2% (Electron microscopy grade, Sigma #G5882) and post-fixed in 2% OsO4 in 0.1M cacodylate buffer (pH 7.2), serially dehydrated in increasing ethanol concentrations, embedded in Agar 100 resin (Agar Scientific Ltd, UK) and left to polymerize at 60°C for 2 days. Ultrathin sections (50-70 nm thick) were produced with a Leica EM UC6 ultra-microtome, collected on formvar-carbon-coated copper grids, and stained with uranyl acetate and lead citrate by standard procedures. Observations were made on a Tecnai 10 TEM (FEI) and images were captured with a Veleta CCD camera and processed with SIS iTEM (Olympus). Quantification of the dimensions of the most apical desmosome in organoids was performed using the NDPview2 software. For SEM, samples were fixed over night at 4°C in glutaraldehyde 2.5%, 0.1M cacodylate buffer (pH 7.2), and post-fixed in OsO4 (2%) in the same buffer. After serial dehydration, samples were dried at critical point and coated with platinum by standard procedures. Observations were made in a Tecnai FEG ESEM QUANTA 200 (FEI) and images processed by SIS iTEM (Olympus) software. Quantification of organoid crackles/mm2 was performed using the Image J software. Quantification was done on 4 CDEO and 3 MDEO lines, with 34-45 pictures being analyzed per organoid line.

***RNA extraction, RNA sequencing and Gene Set Enrichment Analysis (GSEA)***

Organoid samples were collected for bulk RNA sequencing at the indicated passage (p) and day (d): CDEO1 (p1d35), CDEO3 (p2d20), CDEO4 (p2d26), CDEO6 (p2d15), CDEO7 (p2d21), CDEO9 (p2d25), CDEO11 (p2d21), MDEO1 (p2d16), MDEO2 (p2d16), MDEO5 (p2d17), MDEO8 (p3d33), MDEO9 (p1d25), MDEO12 (p4d19), MDEO13 (p4d19). Total RNA from biopsies and organoids was extracted using the miRNA isolation kit (Ambion, Life Technologies, AM1560). Following RNA quality check using a Fragment analyzer 5200 (Agilent technologies), RNAs extracted from organoids (7 CDEOs and 7 MDEOs) and biopsies (12 controls and 13 MASH) were then processed to obtain indexed cDNA libraries using the NEBNext Ultra II directional RNA Library Prep Kit for Illumina® E7760L (NEW ENGLAND BioLabs Inc) following manufacturer recommendations. The multiplexed libraries were loaded onto a NovaSeq 6000 (Illumina) using an S2 flow cell and sequences were produced using a 200 Cycles Kit. Sequenced reads were trimmed for adaptor sequence (Trimmomatic-0.36). Paired-end reads were mapped against the human reference genome (GRCm38.p4/mm10) using the STAR_2.5.3a software to generate read alignments for each sample. After transcripts assembling, gene-level counts were obtained using HTSeq-0.9.1.

***Gene expression analysis by qRT-PCR***

qRT-PCR was performed on total RNA extracted from organoid cultures using the miRNA isolation kit. A DNAse I treatment (Invitrogen) was used to remove potential contaminant DNA. cDNA was prepared using RnaseOUT and Superscript II according to the manufacturer’s protocol (Invitrogen). qPCRs were performed on the qTower 3 from Analytik Jena. Gene expression levels were normalized to that of reference genes (GAPDH, YWHAZ) and quantified using the qBase Software (CellCarta). Primer sequences are reported in the Table 1-reagents.

***Transepithelial electrical resistance measurements and paracellular permeability assay***

Calcium depletion assays were performed on fully confluent cultures (reached between day 13-15) by adding 2 mM EGTA in HBSS buffer for 30 minutes. Then, after wash with DPBS, cells were cultured in complete medium (containing 1.05 mM calcium chloride) for a further 150 minutes. Read data were corrected by subtracting the value of the blank corresponding to Matrigel-precoated wells containing no organoids and finally expressed as ohm.cm^2^. Experiments were repeated at least twice for each organoid line, with 3 measures/well at any time point for each well. Paracellular permeability was determined by measuring the diffusion of FITC-Dextran 4 kDa, initially added apically at 0.5 mg/ml to the culture medium, on the basolateral pole after 24 hours. Fluorescence was detected with the Microwin software on Mithras LB940 reader (Berthold Technologies) at 528 nm. Wells precoated with 1 % matrigel without cells were used as controls.

***TABLE 1-Reagents***

| ***Cell lines*** | ***Resource Reference/source Identifier or catalog number*** |  |
| --- | --- | --- |
| L-Wnt3a cells from Mus musculus | ATCC / CRL-2647 |  |
| ***Antibodies/probes*** | ***Resource Reference/source Identifier or catalog number*** | ***Dilution*** |
| Mouse anti-CDX2 | BioGenex AM-392-5M | 1/100 |
| Mouse anti-CDH17 | R & D systems/ MAB1032 | 1/100 |
| Mouse anti-DCS2 | Invitrogen/ 32-6200 | 1/100 |
| Rabbit anti-CLDN2 | Proteintech/ 26912-1-AP | 1/200 |
| Rabbit anti-EPCAM | Abcam/ ab71916 | 1/200 |
| Rabbit anti-OLFM4 | Cell signaling/ 14369T | 1/100 |
| Rabbit anti-LYSOZYME | Dako/ PA5-22072 | 1/100 |
| Mouse anti-OCLN | Proteintech/ 66378-1-Ig | 1/200 |
| Rabbit anti-VILLIN | Invitrogen/ PA5-22072 | 1/500 |
| Rat anti-ZO1 | Invitrogen/ 402200 | 1/100 |
| Rabbit anti-KI67 | Abcam/ ab15580 | 1/100 |
| GS-II lectin-AF647 | Molecular Probes/ L-32451 | 1/100 |
| UEA-I lectin-Biotinylated | GALAB technology/ 150141 | 1/1000 |
| Donkey anti-mouse-Cy3 | Jackson Immunoresearch / 715-165-150 | 1/500 |
| Donkey anti-rabbit-AF488 | Jackson Immunoresearch / 711-545-152 | 1/500 |
| Donkey anti-rabbit-biotinylated | Jackson Immunoresearch / 711-065-152 | 1/500 |
| Dapi | Sigma Aldrich/D9542 | 1/2,000 |
| Phalloidin-FITC | R &D systems/P582 | 1/300 |
| human CLDN2 RNAscope | ACD-Biotechne/ 492051 |  |
| FITC-Dextran 4 kDa | Sigma/ FD4 | 0.5 mg/ml apically |
| ***Oligonucleotides for qRT-PCR*** | ***Forward 5'-3'*** | ***Reverse 5'-3'*** |
| hACACA | ACAACGCAGGCATCAGAAGA | GTTTCACCGCACACTGTTCC |
| hACLY | GACTTCGGCAGAGGTAGAGC | TCTGGATGGCTGAGGTGGTA |
| hCD36 | CGGCTGCAGGTCAACCTATT | CACCAATGGTCCCAGTCTCA |
| hCPT1A | TCCAGTTGGCTTATCGTGGTG | TCCAGAGTCCGATTGATTTTTGC |
| hDGAT2 | CCCTCATAGCCGCCTACTCC | CAGTGCCCCATCTCCCAGA |
| hFABP1 | GTGTCGGAAATCGTGCAGAAT | GACTTTCTCCCCTGTCATTGTC |
| hFASN | CAACCTCTCCCAGGTATGCG | CCAGGGAGCTGTGGATGATG |
| hGAPDH | CGCTCTCTGCTCCTCCTGTT | CCATGGTGTCTGAGCGATGT |
| hHMGCS2 | GACTCCAGTGAAGCGCATTCT | CTGGGAAGTAGACCTCCAGG |
| hLGR5 | CACACACTGTCATTGCGAGC | GTGAAGACGCTGAGGTTGGA |
| hOLFM4 | GAGGTTCTGTGTCCCAGTTGT | CAAGCGTTCCACTCTGTCCA |
| hSCD | ACACCCAGCTGTCAAAGAGA | GCCAGGTTTGTAGTACCTCCTC |
| hSCL27A1 | GTACCACTCGGCAGGAAACA | ACTTGATGCAGTCGTCCCAG |
| hYWHAZ | ACTTTTGGTACATTGTGGCTTCAA | CCGCCAGGACAAACCAGTAT |
| ***Chemicals, enzymes and other reagents*** | ***Resource Reference/source Identifier or catalog number*** |  |
| Formalin solution, buffered | Sigma Aldrich/ HT5011 | 10% |
| Sucrose | Millipore/ 1076511000 | 20-30 % w/v (tissue embedding); 1 % w/v (chelating buffer) |
| Sorbitol | Sigma Aldrich/ S1876 | 2 % w/v (chelating buffer) |
| Bovine serum albumin | Sigma Aldrich/ A3294 | 1 % w/v (chelating buffer) |
| Tissue freezing medium | Leica/ 14020108926 |  |
| Sodium citrate (pH 6.0) | VWR/ 27833.294 | 10 mM |
| Triton X100 | Sigma Aldrich/ T8787 | 0.1% (v/v) |
| Horse serum | ThermoFisher/ 16050122 | 5% (v/v) |
| Glutaraldehyde, electron microscopy grade | Sigma Aldrich/ G5882 | 2 % or 2.5 % (v/v) |
| OsO4, electron microscopy grade | Electron microscopy science/ 20816-12-0 | 2% |
| Vectastain elite ABS kit, peroxidase | Vector laboratories/PK-6100 |  |
| DAB substrate kit | Vector laboratories/ SK-4100 |  |
| Mayers' hemalun solution | Millipore/ 1092492500 |  |
| Coverquick 4000 | VWR Chemicals/ 5547539 |  |
| Alcian blue 8GX | Sigma-Aldrich/ A3157 |  |
| Nuclear fast red | Sigma-Aldrich/ 229113 |  |
| Fluorsave reagent | Millipore/ 345789 |  |
| miRNA isolation kit | Ambion life technologies/ AM1560 |  |
| NEB Next ultra II directional RNA library pre kit | Illumina/ E7760L |  |
| ***Cell culture reagents*** | ***Resource Reference/source Identifier or catalog number*** | ***Final concentration*** |
| Advanced-DMEM/F12 medium | Thermo fisher scientific/ 12634028 | 1 X |
| DMEM | Gibco/41965-039 | 1 X |
| Wnt3a-conditioned medium | ATCC L-Wnt3a CRL2647 | 50% |
| GlutaMAX | Thermo fisher scientific/ 35050061 | 2 mM |
| N2 | Thermo fischer scientific/ 17502048 | 1 X |
| B27 w/o vit.A | Thermo fisher scientific/ 12587010 | 1 X |
| Amphotericin | Thermo fisher scientific/ 15290026 | 2.5 µg/ml |
| Gentamycin | Thermo fisher scientific/ 15750037 | 40 µg/ml |
| penicillin-streptomycin cocktail 100 X | Thermo fisher scientific/ 15070063 | 5 U/ml |
| UltraPure EDTA 0.5mM, pH = 8 | Invitrogen 15575038 | 2 mM |
| HEPES | Thermo fisher scientific/ 15630080 | 10 mM |
| N acetyl cysteine | Sigma Aldrich/ A9165 | 1 mM |
| human EGF | Peprotech/ 100-15 | 50 ng/ml |
| human Noggin | Peprotech/ 120-10C | 100 ng/ml |
| human Rspondin 1 | Peprotech/ 120-38 | 1 µg/ml |
| Nicotinamide | Sigma Aldrich/ N0636 | 10 mM |
| Gastrin | Sigma Aldrich/SCP0152 | 10 nM |
| A8301 | Sigma Aldrich/SML0788 | 500 nM |
| SB202190 | Abcam/ ab120638 | 10 µM |
| TryplExpress | Thermo fisher scientific/ 12605028 | Ready-to-use |
| HBSS | Thermo fisher scientific/ 14175095 | Ready-to-use |
| DPBS | Thermo fisher scientific/14190094 | 1 X |
| Basement membrane matrix, Growth Factor Reduced (GFR) Basement Membrane Matrix, Growth factor reduced, Basement membrane matrix, Phenol Red-free, LDEV-free | Corning/ 356231 | 100% |
| CHIR99021 | StemGent/ 040004 | 3.6 µM |
| Thiazovivin | Sigma Aldrich/ SML1045 | 2.5 µM |
| Y-27632 | Sigma Aldrich/Y0503 | 10 µM |
| 100 µm cell strainer | VWR 10054458 | / |
| Fetal bovine serum (FBS) | ThermoFisher 10270106 | / |
| Transwell with inserts | Dulis/ 003470 |  |
| Oleic acid | Sigma Aldrich/ O1008 | 300 mM stock solution containing  2 mM BSA |
| Palmitic acid | Sigma Aldrich/ P0500 | 250 mM stock solution containing 2 mM BSA |
| ***Softwares*** | ***Resource Reference/source Identifier or catalog number*** |  |
| GraphPad Prism 10 | https://www.graphpad.com |  |
| qPCR4.0 | Analytik Jena |  |
| qBase | Biogazelle |  |
| GSEA MolSig | Broad Institute |  |
| Degust | Monash Institute |  |
| ZEN Blue 3.5 | Zeiss |  |
| NDP.view2 | Hamamatsu |  |
| Biorender | https://www.biorender.com/ |  |
| Image J |  |  |
| ***Other Resources*** | ***Reference/source Identifier or catalog number*** |  |
| Nanozoomer digital scanner | Hamamatsu |  |
| Axio Observer inverted microscope | Zeiss |  |
| AE31 microscope/Moticam Pro camera | Motic |  |
| Fragment Analyzer 5200 | Agilent technologies |  |
| NovaSeq 6000 | Illumina |  |
| EVOM^TM^ device for TEER measurement | Word precision Instruments |  |

***SUPPLEMENTARY FIGURES***

**
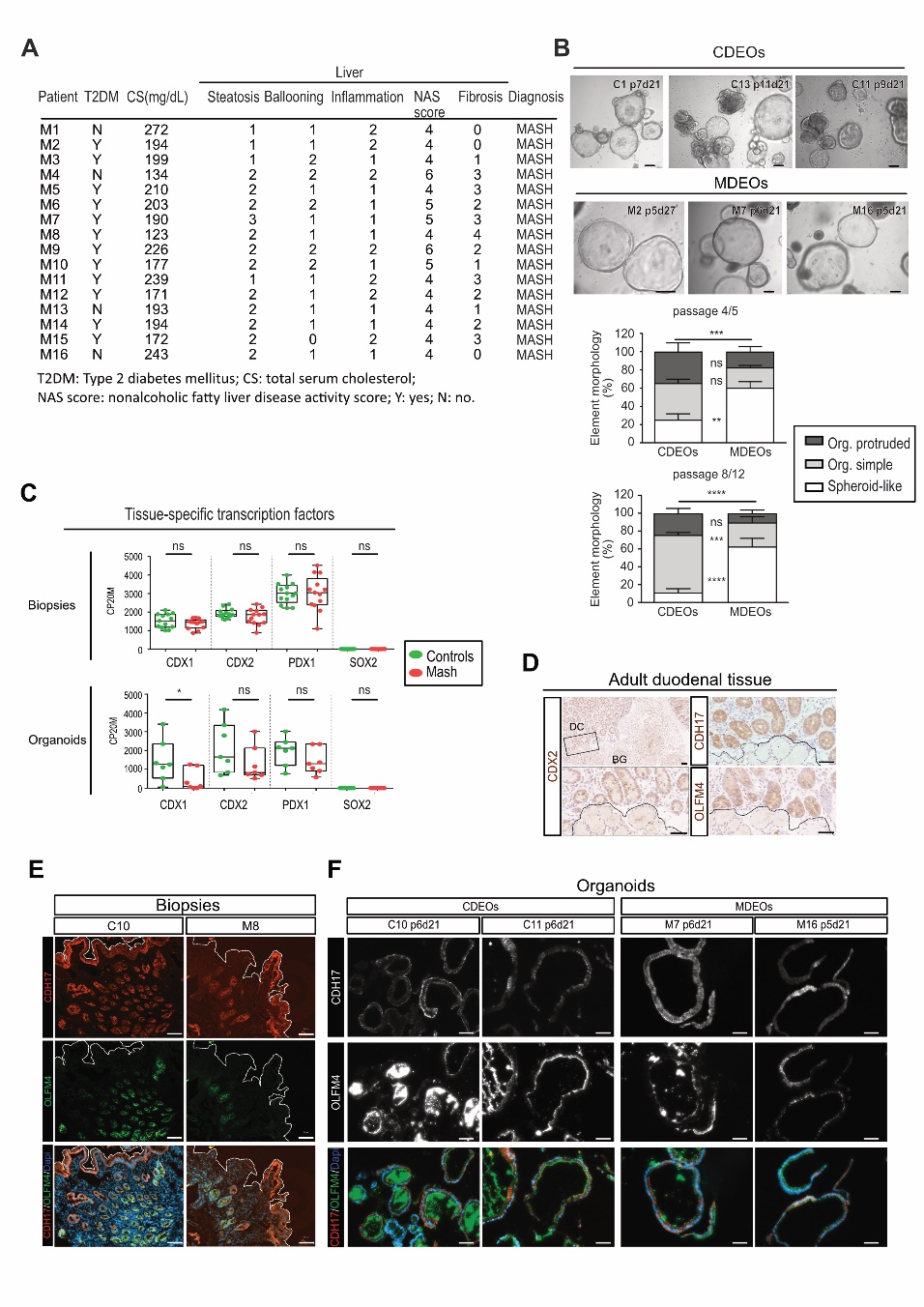
**

**Figure S1. Generation of a living duodenal organoid biobank from human MASH patients.**

**A.** Table showing some biological parameters of MASH patients. **B.** Representative pictures of various CDEO and MDEO lines at advanced passage. Identity, passage, and day of culture are indicated for each organoid line. Scale bars: 150 µm. Lower panels: quantification of organoid complexity was determined at passages 4/5 (for n=6 CDEO and 6 MDEO lines) and passages 8/12 (for n=4 CDEO and 4 MDEO lines). Org: organoid. Categories are expressed as the mean ± sem. Passage 4/5: two-way ANOVA (interaction *** P< 0.001) followed by Sidak's multiple comparisons test: MDEO vs CDEO spheroids: **P< 0.001; MDEO vs CDEO organoid simple or organoid protruded: ns, not significant. Passage 8/12: two-way ANOVA (interaction **** P< 0.001) followed by Sidak's multiple comparisons test: MDEO vs CDEO spheroids: ****P< 0.0001; MDEO vs CDEO organoid simple: ***P= 0.0006; MDEO vs CDEO organoid protruded: ns, not significant. **C.** Expression levels of genes coding for tissue-specific transcription factors. CP20M: counts per kilobase of transcript per 20 million mapped reads. Each symbol corresponds to the value of biopsy or organoid line obtained from an individual subject. Mann-Whitney test: *P< 0.01; ns, not significant. **D**. Immunohistochemistry showing expression of CDX2 and CDH17 in the duodenal epithelium and expression of the stem cell marker OLFM4 restricted to the duodenal crypts (DC). Brunner’s glands are delineated as BG. Scale bars: 50 µm. **E.** Immunofluorescence showing CDH17 and OLFM4 expression in biopsies. Nuclei counterstained with DAPI (merge image). The dotted line delineates the limit of the epithelium. Scale bars: 50 µm. **F.** Immunofluorescence showing CDH17 and OLFM4 expression in CDEO and MDEO lines. Identity and day of culture for each organoid line are indicated. Nuclei counterstained with DAPI (merge image). Scale bars: 50 µm.

**
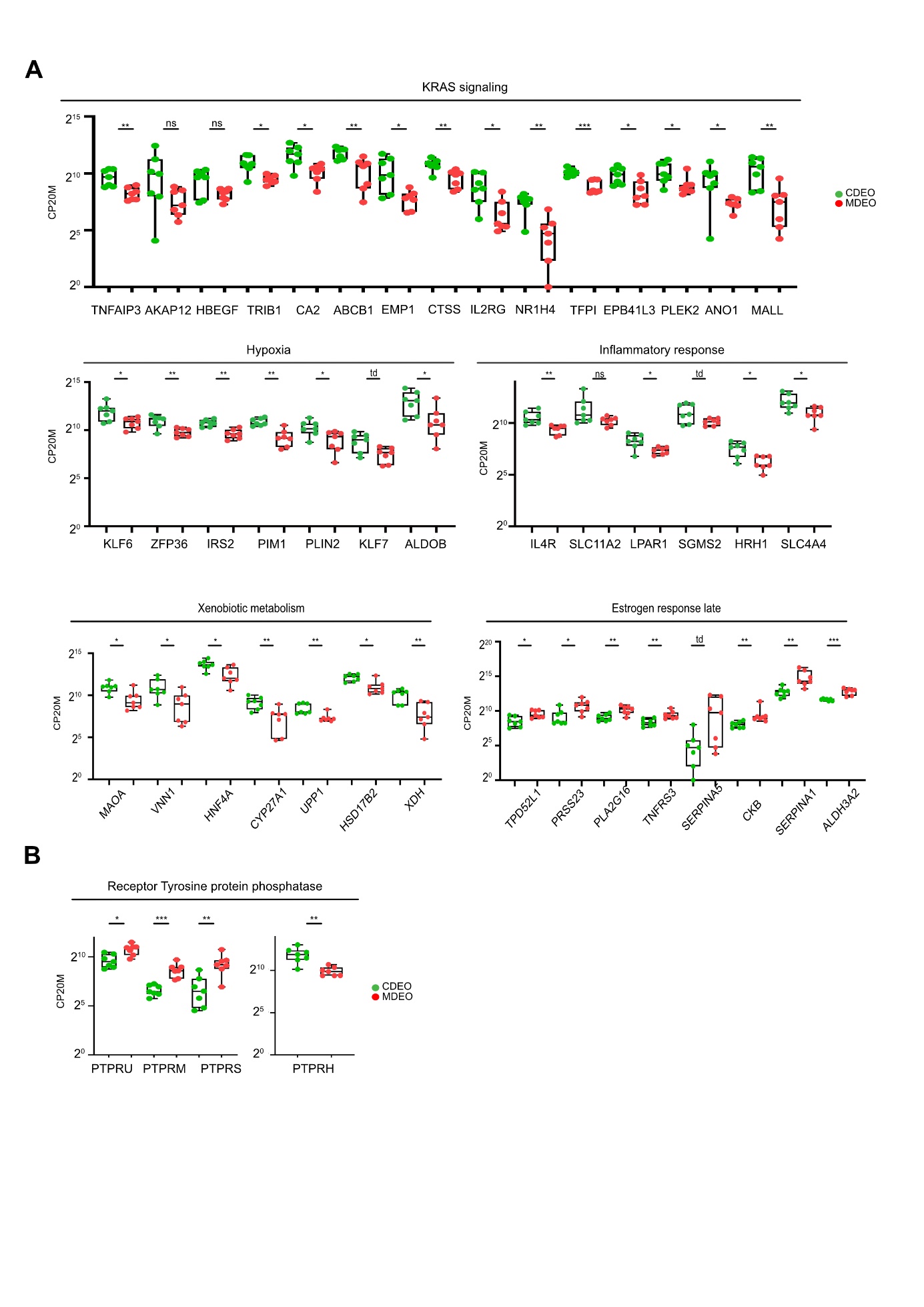
**

**Figure S2. Altered cell signaling pathways in MDEOS vs CDEOs.**

**A.** Expression levels of genes involved in cell signaling processes. CP20M: counts per kilobase of transcript per 20 million mapped reads. Each symbol corresponds to the value of an organoid line obtained from an individual subject. Mann-Whitney test: ***P < 0.001; **P < 0.01; *P < 0.05; td: P<0.1. **B**. Expression levels of genes coding for tyrosine protein phosphatases. CP20M: counts per kilobase of transcript per 20 million mapped reads. Each symbol corresponds to the value of an organoid line obtained from an individual subject. Mann-Whitney test: ***P < 0.001; **P < 0.01; *P < 0.05.

**
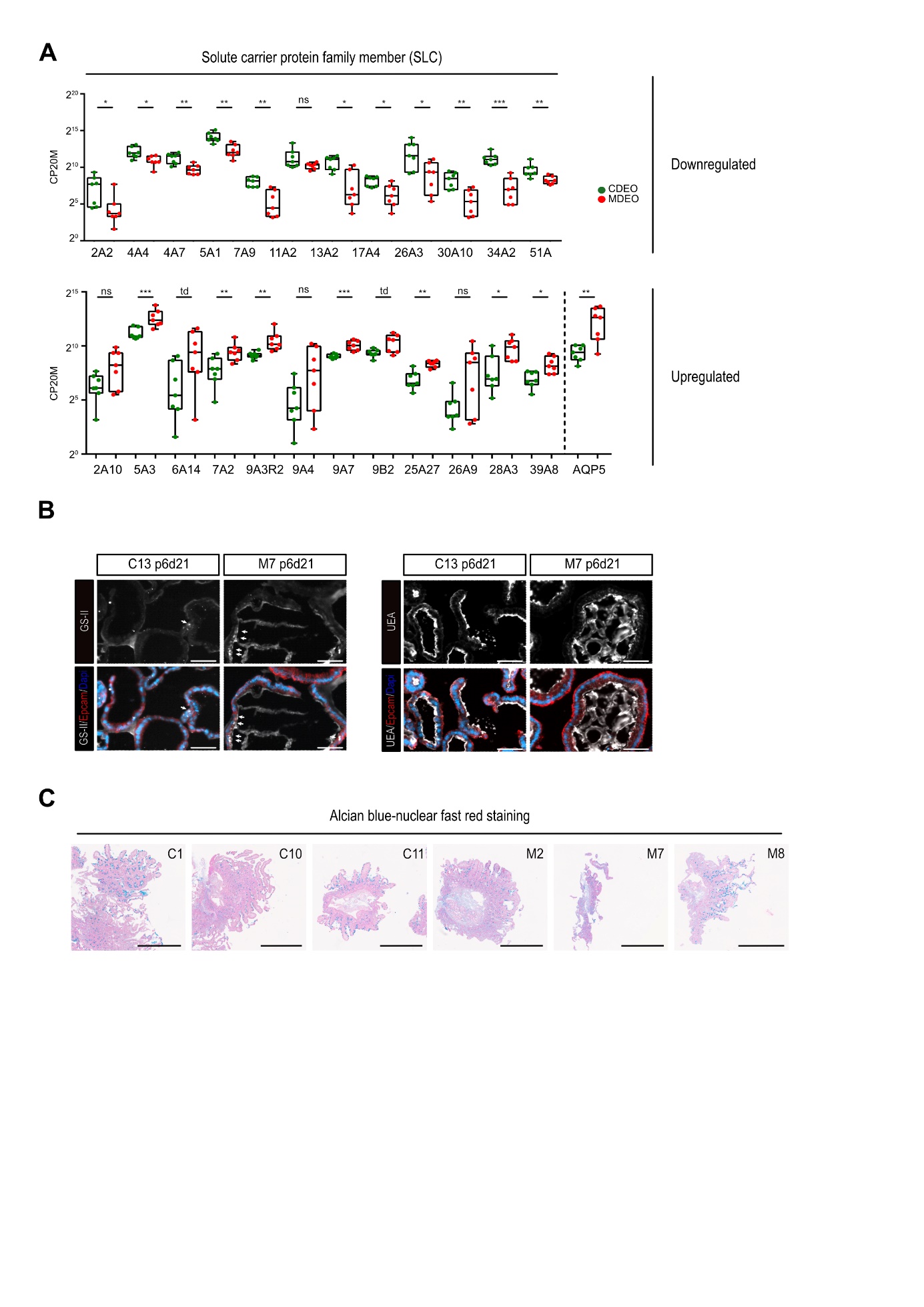
**

**Figure S3. Transcriptome analysis of MASH-derived organoids reveals altered homeostasis.**

**A.** Expression levels of genes coding for differentially expressed solute carrier protein family members. CP20M: counts per kilobase of transcript per 20 million mapped reads. Each symbol corresponds to the value of an organoid line obtained from an individual subject. Mann-Whitney test: ***P < 0.001; **P < 0.01; *P < 0.05; td (tendency): P<0.1; ns: not significant. **B.** Immunofluorescence showing altered expression of mucins in MDEOs vs CDEOs using GS-II and UEA-I lectins to label MUC6 and MUC2-expressing cells, respectively. Cell membranes shown with EPCAM and nuclei counterstained with DAPI. Arrows indicate cells labelled with GS-II. Scale bars: 50 μm. **C.** Alcian blue-nuclear fast red staining visualizing acid mucins in duodenal biopsies. Scale bars: 1 mm.

**
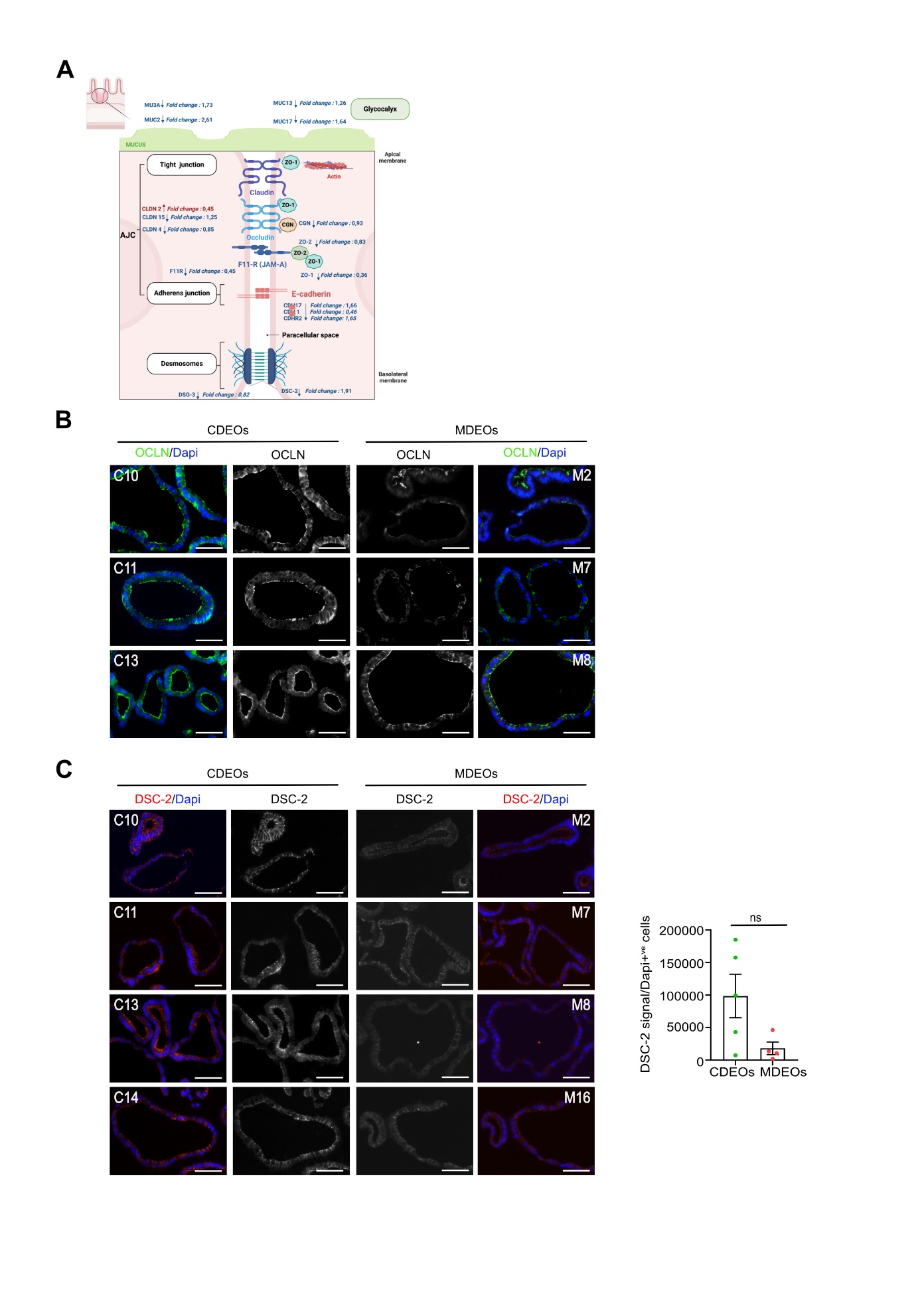
**

**Figure S4. Transcriptome analysis of MASH-derived organoids reveals dysregulated expression of cell junction components.**

**A.** Schematic representation showing differentially modulated genes involved in cell adhesion. Log 2-fold change in MDEOs vs CDEOs is indicated. **B.** Representative pictures of immunofluorescence showing predominant apical localization of OCLN in organoids. Nuclei counterstained with Dapi. **C.** Representative pictures of immunofluorescence showing basolateral localization of DSC2 in organoids. Nuclei counterstained with Dapi. Right panel: quantification of DSC2 expression levels relative to the total number of cells. Each symbol refers to an individual organoid line (n=5 CDEOs, n=4 MDEOs). A mean of 267 cells were analyzed per organoid line. Mann-Whitney test: ns: not significant. Scale bars: 50 µm (B, C).

**
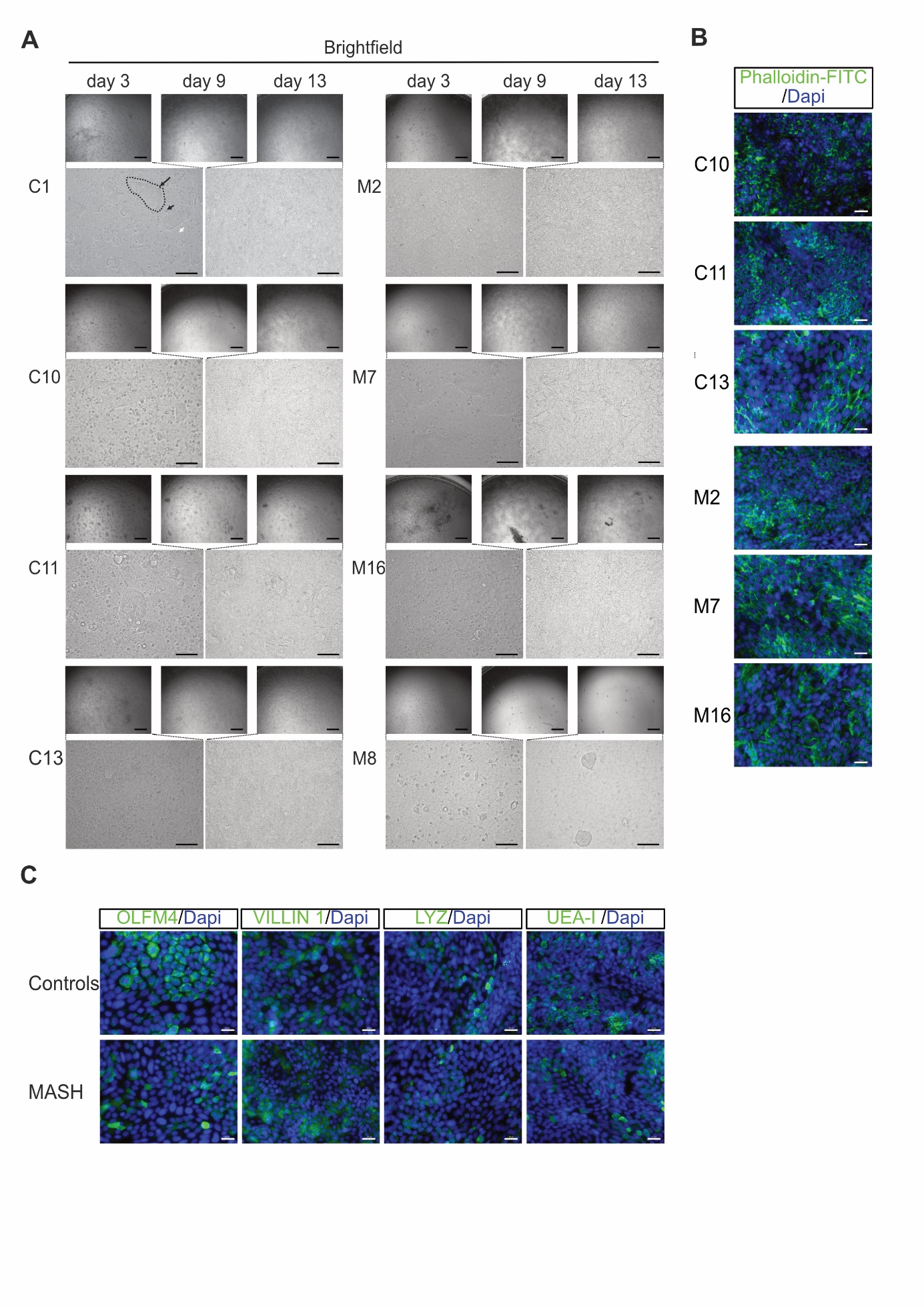
**

**Figure S5. Characterization of the organoid lines grown on transwells.**

**A.** Representative pictures of transwell cultures over time. The long and short black arrows indicate a cell cluster (circled) and isolated cells, respectively. The white arrow points to a region of the transwell devoid of any cell. Scale bars: 500 µm (upper panels) and 200 µm (lower panels). **B**. Representative pictures showing fully confluent monolayers using Phalloidin-FITC. Nuclei were counterstained with Dapi. Scale bars: 20 µm. **C.** Representative pictures showing cell lineage differentiation in monolayers using anti-OLFM4, anti-VILLIN, anti-LYZ and UEA-I lectin. Nuclei were counterstained with Dapi. Scale bars: 20 µm.
